# TssA from *Burkholderia cenocepacia*: expression, purification, crystallization and crystallographic analysis

**DOI:** 10.1107/S2053230X18009706

**Published:** 2018-08-29

**Authors:** Hayley J. Owen, Ruyue Sun, Asma Ahmad, Svetlana E. Sedelnikova, Patrick J. Baker, Mark S. Thomas, David W. Rice

**Affiliations:** a Department of Molecular Biology and Biotechnology, Krebs Institute, University of Sheffield, Western Bank, Sheffield S10 2TN, England; b Department of Infection, Immunity and Cardiovascular Disease, University of Sheffield Medical School, Beech Hill Road, Sheffield S10 2RX, England

**Keywords:** type VI secretion system, TssA, *Burkholderia cenocepacia*

## Abstract

TssA is a core component of the type VI secretion system, and phylogenetic analysis of TssA subunits from different species has suggested that these proteins fall into three distinct clades. Whilst representatives of two clades, TssA1 and TssA2, have been the subjects of investigation, no members of the third clade (TssA3) have been studied. Constructs of TssA from *Burkholderia cenocepacia*, a representative of clade 3, were expressed, purified and subjected to crystallization trials. Data were collected from crystals of constructs of the N-terminal and C-terminal domains. Analysis of the data from the crystals of these constructs and preliminary structure determination indicates that the C-terminal domain forms an assembly of 32 subunits in *D*_16_ symmetry, whereas the N-terminal domain is not involved in subunit assocation.

## Abstract 2

Analysis of the noncrystallographic symmetry of crystals of the C-terminal domain of *Burkholderia cenocepacia* TssA indicates a quaternary structure of 32 subunits in *D*_16_ symmetry.

## 1. Introduction   

The type VI secretion system (T6SS) is composed of 14 core components, TssA–TssM and PAAR, which together form a contractile machine that injects protein effectors into target cells (Pukatzki *et al.*, 2007 ▸; Shalom *et al.*, 2007 ▸; Zheng & Leung, 2007 ▸; Boyer *et al.*, 2009 ▸; Leiman *et al.*, 2009 ▸; Cianfanelli *et al.*, 2016 ▸). The core components form a number of sub­complexes, including the sheath/tube complex, membrane complex and baseplate (Leiman *et al.*, 2009 ▸; Basler *et al.*, 2012 ▸; Shneider *et al.*, 2013 ▸; Brunet *et al.*, 2015 ▸; Durand *et al.*, 2015 ▸). TssB and TssC form the contractile sheath, which surrounds the TssD inner tube (Bönemann *et al.*, 2009 ▸; Leiman *et al.*, 2009 ▸; Basler *et al.*, 2012 ▸; Brunet *et al.*, 2014 ▸). TssI and PAAR sit on top of the tube and may be decorated with covalently or noncovalently attached effector proteins (Pukatzki *et al.*, 2007 ▸; Leiman *et al.*, 2009 ▸; Shneider *et al.*, 2013 ▸). Upon contraction of the sheath against the baseplate, the tube, which can also carry effector proteins, passes through the baseplate and is ejected from the cell (Pukatzki *et al.*, 2006 ▸; Basler *et al.*, 2012 ▸; Silverman *et al.*, 2013 ▸). TssH then acts to recycle the sheath components post-contraction for reassembly in the extended state (Bönemann *et al.*, 2009 ▸; Basler *et al.*, 2012 ▸; Kapitein *et al.*, 2013 ▸). TssJ, TssL and TssM form a complex that anchors the contractile machinery to the cell envelope through interactions with the baseplate (Aschtgen *et al.*, 2010 ▸; Zoued *et al.*, 2013 ▸; Brunet *et al.*, 2015 ▸; Durand *et al.*, 2015 ▸). TssE, TssF, TssG and TssK are components of the baseplate, which serves as a platform for priming of the sheath and tube assembly (Basler *et al.*, 2012 ▸; English *et al.*, 2014 ▸; Brunet *et al.*, 2015 ▸).

Following phylogenetic analysis, TssA proteins have been classified into three clades (TssA1–TssA3; Planamente *et al.*, 2016 ▸), with representative TssAs belonging to clades TssA1 (PA0082 from *Pseudomonas aeruginosa*; P.a TssA1) and TssA2 (Ec042_4540 from enteroaggregative *Escherichia coli*; E.c TssA2) having previously been the subject of biochemical and structural studies (Planamente *et al.*, 2016 ▸; Zoued *et al.*, 2016 ▸). The P.a and E.c TssAs have been shown to interact with the T6SS sheath, inner tube and components of the baseplate, with E.c TssA2 also interacting with the membrane complex (Planamente *et al.*, 2016 ▸; Zoued *et al.*, 2016 ▸). P.a TssA1 is proposed to contain two domains which form a ring structure comprising the C-terminal domain with a flexible peripheral N-terminal domain (Planamente *et al.*, 2016 ▸). Similarly, in E.c TssA2 the C-terminal domain oligomerizes to produce a dodecameric structure of two stacked rings with *D*_6_ symmetry (Zoued *et al.*, 2016 ▸). In E.c TssA2 the N-terminal region is predicted to form arms which extend out from the C-terminal dodecamer core (Zoued *et al.*, 2016 ▸). The structure of part of the E.c TssA2 N-terminal region, Nt2, forms a dimer (Zoued *et al.*, 2016 ▸). Bioinformatic analysis identified a conserved region within both the P.a TssA1 and E.c TssA2 N-terminal domains, referred to as ImpA_N (Planamente *et al.*, 2016 ▸; Zoued *et al.*, 2017 ▸). P.a TssA1 was observed to localize at one end of the sheath, whilst E.c TssA2 was seen to move away from an initial site at the membrane concomitant with sheath polymerization (Planamente *et al.*, 2016 ▸; Zoued *et al.*, 2016 ▸). Based on these observations of P.a TssA1 and some low-level predicted similarities in secondary structure, it was suggested that the TssA1 C-terminal region corresponds to the C-terminal region of the phage T4 baseplate component gp6, thereby identifying P.a TssA1 as a possible baseplate component (Planamente *et al.*, 2016 ▸). On the other hand, E.c TssA2 was predicted to be involved in recruitment of the baseplate assembly to the membrane complex and subsequent polymerization of the TssD tube and TssB/TssC sheath (Zoued *et al.*, 2016 ▸).

*Burkholderia cenocepacia* TssA (B.c TssA), locus tag I35_RS01755, is a member of TssA3, the TssA clade that has yet to be investigated (Planamente *et al.*, 2016 ▸). In this paper, we describe the production of constructs of B.c TssA representing the predicted N-terminal and C-terminal domains. Overexpression of the domains, their purification and their crystallization resulted in the production of diffraction-quality crystals, analysis of which provides preliminary information on their stoichiometry, symmetry and structural organization.

## 2. Materials and methods   

### 2.1. Macromolecule production   

Coding sequences for the predicted N-terminal domain (Nt1) and C-terminal domain (CTD) of B.c TssA (UniProt A0A1V2WLD6) were amplified from *B. cenocepacia* strain H111 (Römling *et al.*, 1994 ▸) by PCR using the appropriate combinations of primers as shown in Table 1 ▸. The DNA products were ligated to either plasmid pET-14b (Novagen; encoding a His_6_ purification tag and a thrombin cleavage site) or pMAL-c5X [NEB; encoding a maltose-binding protein (MBP) solubility tag and a Factor Xa cleavage site], followed by transformation of *E. coli* strain JM83 (Yanisch-Perron *et al.*, 1985 ▸). Construct details are shown in Table 1 ▸.

A B.c His_6_-TssA Nt1 construct was overexpressed in *E. coli* BL21 (DE3) cells (Studier & Moffatt, 1986 ▸) grown in brain heart infusion broth (Becton Dickinson) at 37°C to an OD_600_ of 0.5–0.7, whereupon expression was induced by the addition of 1 m*M* IPTG followed by incubation for a further 2–3 h. B.c His_6_-TssA Nt1 was purified from clarified cell lysate in 50 m*M* Tris pH 8.0, 500 m*M* NaCl, applied onto a HisTrap HP column (GE Healthcare Life Sciences) and eluted with a linear gradient of imidazole (0–350 m*M*) in 50 m*M* Tris pH 8.0, 500 m*M* NaCl. Fractions containing the B.c His_6_-TssA Nt1 protein were concentrated using a 30 kDa molecular-weight cutoff concentrator and washed with 10 m*M* Tris pH 8.0 for crystallization.

The B.c MBP-TssA CTD fusion protein was overproduced in *E. coli* ER2523 cells (NEB Express) grown in Lennox broth containing 0.2% glucose (Lennox, 1955 ▸) at 37°C to an OD_600_ of 0.6–0.7, whereupon protein overexpression was induced with 0.3 m*M* IPTG and incubation of the culture continued at the same temperature for a further 2–3 h. To prepare selenomethionine-incorporated B.c MBP-TssA CTD protein (B.c SeMet-MBP-TssA CTD), 2 × 500 ml of cells were grown as described above and harvested prior to induction. The cell pellets were then resuspended in selenomethionine minimal medium, harvested and the resuspension was added to 2 × 500 ml selenomethionine minimal medium [10.5 g l^−1^ K_2_HPO_4_, 1.0 g l^−1^ (NH_4_)_2_SO_4_, 4.5 g l^−1^ KH_2_PO_4_, 0.5 g l^−1^ trisodium citrate·2H_2_O, 5.0 g l^−1^ glycerol and 0.5 g l^−1^ each of adenine, guanosine, thymine and uracil] supplemented with 1.0 g l^−1^ MgSO_4_·7H_2_O, 4.0 mg l^−1^ thiamine, 100 mg l^−1^ each of l-lysine, l-phenylalanine and l-threonine, 50 mg l^−1^ each of l-isoleucine, l-leucine and l-valine, 40 mg l^−1^ seleno-l-methionine and 2.0 g l^−1^ glucose. Growth was continued for 1 h at 37°C prior to the induction of B.c MBP-TssA CTD expression by the addition of 0.3 m*M* IPTG and subsequent growth at 37°C overnight.

Harvested B.c MBP-TssA CTD- and B.c SeMet-MBP-TssA CTD-overexpressing cells were each resuspended in column-binding buffer (50 m*M* Tris–HCl pH 8.0, 200 m*M* NaCl) and the cells were lysed by sonication. The cell lysate was cleared by centrifugation before being applied onto amylose resin (NEB) equilibrated with column-binding buffer. After washing the resin with column-binding buffer, the bound material was then eluted in the same buffer with the addition of 10 m*M* maltose. Fractions containing the fusion protein were concentrated and then digested with Factor Xa (5 µg per milligram of protein; NEB) in the presence of 2 m*M* CaCl_2_ at room temperature for ∼14 h to release the target protein from MBP. Following cleavage of the MBP, the products were separated by gel filtration on a Superdex 200 column (GE Healthcare Life Sciences) in 50 m*M* Tris–HCl pH 8.0, 500 m*M* NaCl. The B.c TssA CTD protein obtained from gel filtration was applied to a final amylose column to remove any uncleaved B.c MBP-TssA CTD fusion molecules. This step was added to avoid heterogeneity of the sample. The protein was then concentrated and buffer-exchanged into 5 m*M* Tris–HCl pH 8.0, 50 m*M* NaCl for crystallization. The purified B.c TssA CTD protein has an additional four non-native residues (ISHM) at the N-terminus following proteolytic release from the N-terminal MBP solubility tag (Table 1 ▸).

### 2.2. Crystallization   

B.c TssA protein constructs were set down into sitting-drop crystallization trials using a Matrix Hydra II Plus One robot, with 200 nl:200 nl drops and a 50 µl reservoir, or hanging-drop crystallization trials, and stored at 17°C. A selection of crystallization condition suites were used for screening, including JCSG+, ProPlex, PACT, Classics, MPD, AmSO_4_, PEGs and pHClear (Qiagen, Molecular Dimensions). B.c His_6_-TssA Nt1 crystals grew in sitting-drop trial conditions consisting of 0.16 *M* calcium acetate, 0.08 *M* sodium cacodylate buffer pH 6.5, 14.4%(*w*/*v*) PEG 8000, 20%(*v*/*v*) glycerol. B.c TssA CTD produced crystals in sitting-drop trial conditions consisting of 0.1 *M* sodium chloride, 0.1 *M* Tris pH 8.0, 15%(*v*/*v*) ethanol, 5%(*v*/*v*) MPD. B.c SeMet-TssA CTD produced crystals in hanging-drop trial conditions consisting of 1 *M* ammonium sulfate, 2%(*w*/*v*) PEG 3350, 0.1 *M* bis-tris pH 6.0. Crystallization information is shown in Table 2 ▸.

### 2.3. Data collection, processing and analysis   

Data were collected from B.c His_6_-TssA Nt1 crystals [cryoprotected in 0.16 *M* calcium acetate, 0.08 *M* sodium cacodylate buffer pH 6.5, 16.4%(*w*/*v*) PEG 8000, 30%(*v*/*v*) glycerol] on beamline I04-1 at Diamond Light Source. The data were processed to 1.87 Å resolution using the -3daii option in *xia*2 (Collaborative Computational Project, Number 4, 1994 ▸; Evans, 2006 ▸; Kabsch, 2010 ▸; Winter, 2010 ▸) and indicated that the crystals belonged to space group *P*2_1_2_1_2 (unit-cell parameters *a* = 49.7, *b* = 125.4, *c* = 45.5 Å). Data-collection and processing statistics are shown in Table 3 ▸.

Data were collected from B.c TssA CTD crystals [cryoprotected in 0.1 *M* sodium chloride, 0.1 *M* Tris pH 8.0, 15%(*v*/*v*) ethanol, 25%(*v*/*v*) glycerol] on beamline I04 at Diamond Light Source. Data were processed to 3.79 Å resolution using the -3daii option in *xia*2 (Collaborative Computational Project, Number 4, 1994 ▸; Sauter *et al.*, 2004 ▸; Evans, 2006 ▸; Zhang *et al.*, 2006 ▸; Kabsch, 2010 ▸; Winter, 2010 ▸) and indicated that the crystals belonged to space group *I*222. Data-collection and processing statistics are shown in Table 3 ▸. A self-rotation function was calculated on all of the data from crystals of B.c TssA CTD using an integration radius of 44 Å in *POLARRFN* (Winn *et al.*, 2011 ▸). Self Patterson functions on the native data were calculated using *FFT for Patterson* (Winn *et al.*, 2011 ▸).

B.c His_6_-TssA Nt1 crystals were subjected to the sub­limination of elemental iodine for ∼3 h prior to cryoprotection in 0.16 *M* calcium acetate, 0.08 *M* sodium cacodylate buffer pH 6.5, 16.4%(*w*/*v*) PEG 8000, 30%(*v*/*v*) glycerol and data collection on beamline I03 at Diamond Light Source. The data were processed to 2.01 Å resolution with *fast_dp* (Winter & McAuley, 2011 ▸) and indicated that the crystals belonged to space group *P*2_1_2_1_2 (unit-cell parameters *a* = 45.6, *b* = 49.8, *c* = 126.1 Å). Data-collection statistics are shown in Table 3 ▸.

Data were collected from B.c SeMet-TssA CTD crystals [cryoprotected in 1–1.2 *M* ammonium sulfate, 3–4%(*w*/*v*) PEG 3350, 0.1 *M* bis-tris pH 6.0, 30%(*v*/*v*) glycerol] on beamline I03 at Diamond Light Source. The data were processed to 2.86 Å resolution using the -3d option in *xia*2 (Collaborative Computational Project, Number 4, 1994 ▸; Evans, 2006 ▸; Kabsch, 2010 ▸; Winter, 2010 ▸) and indicated that the crystals belonged to space group *I*222 (unit-cell parameters *a* = 46.3, *b* = 201.3, *c* = 263.9 Å). Data-collection statistics are shown in Table 3 ▸.

## 3. Results and discussion   

### 3.1. Construct design   

**Figure 1:**
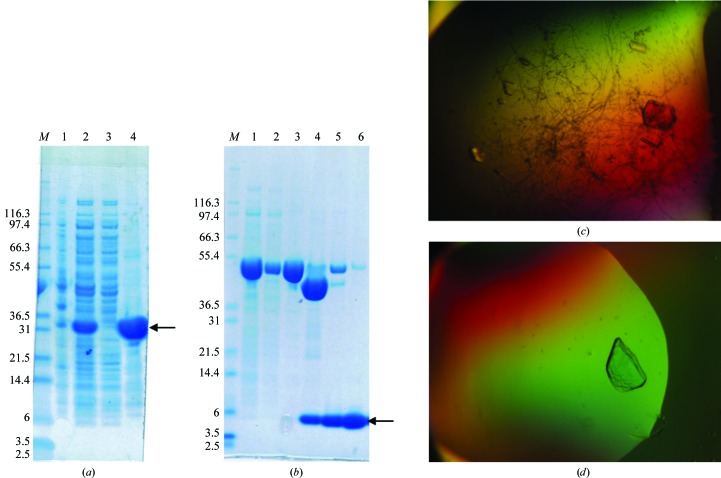
(*a*) SDS–PAGE analysis of protein purification of the B.c His_6_-TssA Nt1 construct. Lane *M*, Mark12 marker (labelled in kDa). Lane 1, cell debris. Lane 2, cell-free extract. Lane 3, flowthrough (Ni column). Lane 4, final preparation. (*b*) SDS–PAGE analysis of protein purification of the B.c SeMet MBP-TssA CTD construct. The protein, indicated by the arrow, is shown to be ∼90% pure in the final preparation samples and thus was used for crystallization. Lane *M*, Mark12 marker (labelled in kDa). Lane 1, cell-free extract. Lane 2, unbound material (amylose column). Lane 3, B.c SeMet MBP-TssA CTD. Lane 4, after MBP cleavage. Lane 5, after gel filtration. Lane 6, final preparation. (*c*) B.c His_6_-TssA Nt1 crystals grown in 0.16 *M* calcium acetate, 0.08 *M* sodium cacodylate buffer pH 6.5, 14.4%(*w*/*v*) PEG 8000, 20%(*v*/*v*) glycerol. (*d*) B.c TssA CTD crystals grown in 0.1 *M* sodium chloride, 0.1 *M* Tris pH 8.0, 15%(*v*/*v*) ethanol, 5%(*v*/*v*) MPD.

The design of the constructs for the N- and C-terminal domains of B.c TssA was based on an analysis of the pattern of sequence conservation across the different clades of TssA and resulted in stable, soluble samples of the proteins encoded by these regions of the gene. Constructs of the predicted N-terminal domain (Nt1) and C-terminal domain (CTD) of B.c TssA produced crystals (Fig. 1 ▸) which diffracted to high resolution.

### 3.2. TssA data analysis   

**Figure 2:**
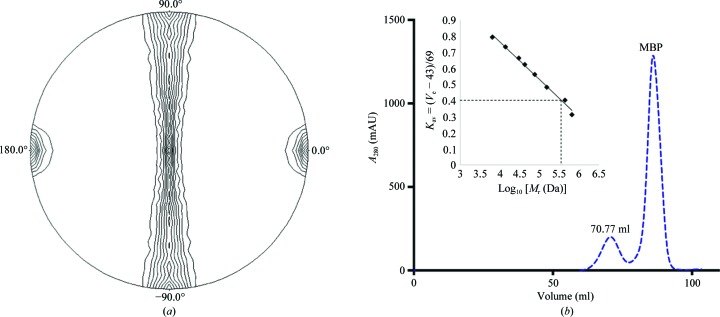
(*a*) The self-rotation function for B.c TssA CTD crystals calculated on all data from 55.90 to 3.79 Å resolution using a 44 Å radius of integration. The section shown corresponds to κ = 180°, with the orthogonal *x*, *y* and *z* axes along the crystallographic *a*, *b* and *c* axes, respectively. Noncrystallographic twofold axes can be seen every 11.25° in the *bc* plane at approximately 70% of the origin. The image was generated in *POLARRFN* (Winn *et al.*, 2011 ▸). (*b*) Gel-filtration chromatogram of B.c SeMet-MBP-TssA CTD protein, with UV absorption (280 nm) shown as a dotted blue line and the elution volume (70.77 ml) corresponding to B.c TssA CTD shown above the curve. Inset: calibration curve for the gel-filtration column, with *K*_av_ = 0.4 (*V*_e_ = 70.77 ml) highlighted corresponding to a molecular mass of ∼3.55 × 10^5^ Da for B.c TssA CTD.

**Figure 3:**
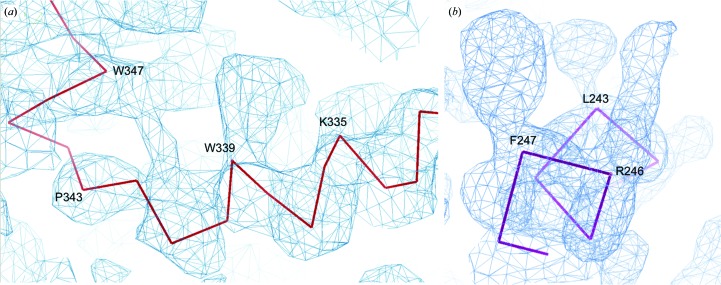
Preliminary electron-density maps, contoured at ∼1.0σ, representing (*a*) a helical section of B.c TssA CTD. The large side chain visible towards the C-­terminal end of the helix corresponds to Trp339, and density can also be seen for the nearby Trp347. Phases were obtained from *SHELX* (Sheldrick, 2008 ▸). (*b*) A helical section of the B.c His_6_-TssA Nt1 domain. The large side chain visible at the C-terminal end of the helix corresponds to Phe247, and density can also be seen for the side chain of the adjacent Arg246. Phases were obtained from *fast_ep* and *SHELX* (Sheldrick, 2008 ▸). Images were generated in *Coot* (Emsley *et al.*, 2010 ▸).

Analysis of the Matthews coefficient of the B.c His_6_-TssA Nt1 crystals suggests that the crystals contain one molecule of ∼30.7 kDa in the asymmetric unit, with a *V*_M_ of 2.31 Å^3^ Da^−1^ (Matthews, 1968 ▸; Kantardjieff & Rupp, 2003 ▸; Winn *et al.*, 2011 ▸). In contrast, the contents of the asymmetric unit of the B.c TssA CTD crystals could not be confidently assigned from analysis of the Matthews coefficient. Consideration of possible values of *V*_M_ suggests that the asymmetric unit contains between nine and 21 subunits (4.13–1.77 Å^3^ Da^−1^; Matthews, 1968 ▸; Kantardjieff & Rupp, 2003 ▸; Winn *et al.*, 2011 ▸). It has previously been reported that the TssA CTD acts as an oligomerization domain which produces ring-like or star-like structures, as has been shown for P.a TssA1 and E.c TssA2, respectively (Planamente *et al.*, 2016 ▸; Zoued *et al.*, 2016 ▸). Self Pattersons calculated using the native data to 6 or 3.79 Å resolution showed no significant peaks above 15% of the origin, consistent with the absence of translational symmetry in the crystal. A self-rotation function was calculated on the B.c TssA CTD data using an integration radius of 44 Å to detect the presence of noncrystallographic symmetry. This identified peaks on the κ = 180° section with a height of approximately 70% of the origin corresponding to twofold axes located in the *bc* plane every 11.25° (Fig. 2 ▸). Consideration of the rotational symmetry and apparent molecular weight, determined by gel-filtration analysis (Fig. 2 ▸), suggests that the quaternary structure of B.c TssA CTD is composed of 32 subunits in *D*_16_ symmetry. This suggests that the asymmetric unit contains eight subunits with an unusually high *V*_M_ (4.64 Å^3^ Da^−1^; Matthews, 1968 ▸; Kantardjieff & Rupp, 2003 ▸; Winn *et al.*, 2011 ▸). A preliminary electron-density map was calculated using SAD data to 2.86 Å resolution from the selenomethionine derivative of the B.c TssA CTD crystals, with an initial chain trace confirming the presence of B.c TssA CTD in the crystals and the nature of the symmetry indicated by the self-rotation function (Fig. 2 ▸). Similarly, a preliminary electron-density map has been calculated using SAD data to 2.01 Å resolution from iodine-exposed B.c His_6_-TssA Nt1 crystals (Fig. 3 ▸). Refinement of the structures and attempts to extend the resolution of the data are ongoing.

## Figures and Tables

**Table 1:** Construct information

Construct	His_6_-TssA Nt1	MBP-TssA CTD
B.c TssA amino acids	1–255	303–373
Tag details	MGSSHHHHHHSSGLVPRGSH	(MBP)NSSSNNNNNNNNNNLGIEGR/ISHM†
Construct sequence	MGSSHHHHHHSSGLVPRGSHMPINLPELLTPISEASPSGDDLLFSNEFDAIQDARRYDDPTLDQGEWVTEIKEADWGFVVDHAGELLRTRTKDLRLAVWLTEALALEDGITGLTEGYALLEGLCREFWDTFHPLPEDDDIEHRLGNVAWLSGRTAELLRAVPLTDGASNAFSTLDWEVAQHVAQSIKRDPEHADDIARGKPSIEQIDASRRVTSIAFYTALLANLKAFEFALDAFEERLVERAGDSAPSFRQARDAFETVYRLAERFAREQGYTG	(MBP)NSSSNNNNNNNNNNLGIEGR/ISHMIQNRAQAVDQLRAVARYFRQTEPHSPVAYLADKAAEWADMPLHKWLESVVKDDGSLSHIRELLGVRPDEQS
Forward primer‡	pET-14b-iotAfor, GCGC**CATATG**CCGATCAATCTCCCCGA	pET-14b-ACTDfor3, GCGC**CATATG**ATCCAGAACCGTGCGCAGGC
Reverse primer‡	pET-14b-TssA-NTDrev, GCGC**GGATCC** TTAGCCGGTATAGCCCTGTTCGC	pET-14b-iotArev, GCGC**GGATCC**TGCGTTTACGACTGCTCGTC
Restriction enzymes	NdeI and BamHI	NdeI and BamHI
Expression vector	pET-14b	pMAL-c5X
Antibiotic	100 µg ml^−1^ ampicillin	100 µg ml^−1^ ampicillin
Expression host	*E. coli* BL21 (DE3)	*E. coli* ER2523 (NEB Express)

† MBP is maltose-binding protein; IEGR is the Factor Xa recognition site; / indicates the Factor X cleavage site. Tags are highlighted as underlined regions of the construct sequences.

‡ Bold sequences indicate restriction sites. Translation initiation and termination codons are underlined.

**Table 2:** Crystallization

Construct	His_6_-TssA Nt1	TssA CTD	SeMet-TssA CTD
Method	Sitting-drop vapour diffusion	Sitting-drop vapour diffusion	Hanging-drop vapour diffusion
Composition of reservoir solution	0.16 *M* calcium acetate, 0.08 *M* sodium cacodylate buffer pH 6.5, 14.4%(*w*/*v*) PEG 8000, 20%(*v*/*v*) glycerol	0.1 *M* sodium chloride, 0.1 *M* Tris pH 8.0, 15%(*v*/*v*) ethanol, 5%(*v*/*v*) MPD	1 *M* ammonium sulfate, 2%(*w*/*v*) PEG 3350, 0.1 *M* bis-tris pH 6.0
Volume and ratio of drop	200 nl:200 nl	200 nl:200 nl	2 µl:1 µl (protein:reservoir)
Volume of reservoir (µl)	50	50	1000

**Table 3:** Data-collection and processing statistics Values in parentheses are for the highest resolution shell.

Construct	His_6_-TssA Nt1	His_6_-TssA Nt1 + iodine	TssA CTD	SeMet-TssA CTD
Wavelength (Å)	0.92000	1.70000	0.97949	0.97922
Beamline	I04-1	I03	I04	I03
Detector	PILATUS 2M	PILATUS 6M	PILATUS 6M-F	PILATUS3 6M
Rotation range per image (°)	0.2	0.2	0.2	0.1
Total rotation range (°)	90	720	180	360
Exposure time per image (s)	0.2	0.2	0.2	0.05
Data-processing package	*xia*2 -3daii	*fast_dp*	*xia*2 -3daii	*xia*2 -3d
Space group	*P*2_1_2_1_2	*P*2_1_2_1_2	*I*222	*I*222
*a*, *b*, *c* (Å)	49.7, 125.4, 45.5	45.6, 49.8, 126.1	46.9, 203.0, 267.9	46.3, 201.3, 263.9
α, β, γ (°)	90.0, 90.0, 90.0	90.0, 90.0, 90.0	90.0, 90.0, 90.0	90.0, 90.0, 90.0
Resolution range (Å)	62.69–1.87 (1.92–1.87)	29.68–2.01 (2.06–2.01)	55.90–3.79 (3.89–3.79)	80.61–2.86 (2.93–2.86)
*R*_merge_	0.067 (0.495)	0.131 (0.761)	0.221 (0.871)	0.207 (2.520)
*R*_p.i.m._	0.051 (0.372)	0.031 (0.174)	0.112 (0.487)	0.064 (0.747)
〈*I*/σ(*I*)〉	10.8 (2.0)	20.6 (3.4)	7.6 (2.0)	11.1 (1.1)
Completeness (%)	98.4 (99.3)	98.4 (79.0)	99.4 (99.7)	100.0 (100.0)
Multiplicity	3.3 (3.3)	23.0 (18.7)	5.7 (5.3)	13.0 (13.2)
Total reflections	77962	448717	75744	382400
Unique reflections	23839	19523	13207	29304

## References

[bb1] Aschtgen, M.-S., Gavioli, M., Dessen, A., Lloubès, R. & Cascales, E. (2010). *Mol. Microbiol.* **75**, 886–899. 10.1111/j.1365-2958.2009.07028.x 20487285

[bb2] Basler, M., Pilhofer, M., Henderson, G. P., Jensen, G. J. & Mekalanos, J. J. (2012). *Nature (London)*, **483**, 182–186. 10.1038/nature10846 PMC3527127 22367545

[bb3] Bönemann, G., Pietrosiuk, A., Diemand, A., Zentgraf, H. & Mogk, A. (2009). *EMBO J.* **28**, 315–325. 10.1038/emboj.2008.269 PMC2646146 19131969

[bb4] Boyer, F., Fichant, G., Berthod, J., Vandenbrouck, Y. & Attree, I. (2009). *BMC Genomics*, **10**, 104. 10.1186/1471-2164-10-104 PMC2660368 19284603

[bb5] Brunet, Y. R., Hénin, J., Celia, H. & Cascales, E. (2014). *EMBO Rep.* **15**, 315–321. 10.1002/embr.201337936 PMC3989698 24488256

[bb6] Brunet, Y. R., Zoued, A., Boyer, F., Douzi, B. & Cascales, E. (2015). *PLOS Genet.* **11**, e1005545. 10.1371/journal.pgen.1005545 PMC4604203 26460929

[bb7] Cianfanelli, F. R., Alcoforado Diniz, J., Guo, M., De Cesare, V., Trost, M. & Coulthurst, S. J. (2016). *PLoS Pathog.* **12**, e1005735. 10.1371/journal.ppat.1005735 PMC4924876 27352036

[bb8] Collaborative Computational Project, Number 4 (1994). *Acta Cryst.* D**50**, 760–763.

[bb9] Durand, E., Nguyen, V. S., Zoued, A., Logger, L., Péhau-Arnaudet, G., Aschtgen, M. S., Spinelli, S., Desmyter, A., Bardiaux, B., Dujeancourt, A., Roussel, A., Cambillau, C., Cascales, E. & Fronzes, R. (2015). *Nature (London)*, **523**, 555–560. 10.1038/nature14667 26200339

[bb10] Emsley, P., Lohkamp, B., Scott, W. G. & Cowtan, K. (2010). *Acta Cryst.* D**66**, 486–501. 10.1107/S0907444910007493 PMC2852313 20383002

[bb11] English, G., Byron, O., Cianfanelli, F. R., Prescott, A. R. & Coulthurst, S. J. (2014). *Biochem. J.* **461**, 291–304. 10.1042/BJ20131426 PMC4072051 24779861

[bb12] Evans, P. (2006). *Acta Cryst.* D**62**, 72–82. 10.1107/S0907444905036693 16369096

[bb13] Kabsch, W. (2010). *Acta Cryst.* D**66**, 125–132. 10.1107/S0907444909047337 PMC2815665 20124692

[bb14] Kantardjieff, K. A. & Rupp, B. (2003). *Protein Sci.* **12**, 1865–1871. 10.1110/ps.0350503 PMC2323984 12930986

[bb15] Kapitein, N., Bönemann, G., Pietrosiuk, A., Seyffer, F., Hausser, I., Locker, J. K. & Mogk, A. (2013). *Mol. Microbiol.* **87**, 1013–1028. 10.1111/mmi.12147 23289512

[bb16] Leiman, P. G., Basler, M., Ramagopal, U. A., Bonanno, J. B., Sauder, J. M., Pukatzki, S., Burley, S. K., Almo, S. C. & Mekalanos, J. J. (2009). *Proc. Natl Acad. Sci. USA*, **106**, 4154–4159. 10.1073/pnas.0813360106 PMC2657435 19251641

[bb40] Lennox, E. S. (1955). *Virology*, **1**, 190–206. 10.1016/0042-6822(55)90016-7 13267987

[bb17] Matthews, B. W. (1968). *J. Mol. Biol.* **33**, 491–497. 10.1016/0022-2836(68)90205-2 5700707

[bb18] Planamente, S., Salih, O., Manoli, E., Albesa-Jové, D., Freemont, P. S. & Filloux, A. (2016). *EMBO J.* **35**, 1613–1627. 10.15252/embj.201694024 PMC4969574 27288401

[bb19] Pukatzki, S., Ma, A. T., Revel, A. T., Sturtevant, D. & Mekalanos, J. J. (2007). *Proc. Natl Acad. Sci. USA*, **104**, 15508–15513. 10.1073/pnas.0706532104 PMC2000545 17873062

[bb20] Pukatzki, S., Ma, A. T., Sturtevant, D., Krastins, B., Sarracino, D., Nelson, W. C., Heidelberg, J. F. & Mekalanos, J. J. (2006). *Proc. Natl Acad. Sci. USA*, **103**, 1528–1533. 10.1073/pnas.0510322103 PMC1345711 16432199

[bb21] Römling, U., Fiedler, B., Bosshammer, J., Grothues, D., Greipel, J., von der Hardt, H. & Tümmler, B. (1994). *J. Infect. Dis.* **170**, 1616–1621. 10.1093/infdis/170.6.1616 7996008

[bb22] Sauter, N. K., Grosse-Kunstleve, R. W. & Adams, P. D. (2004). *J. Appl. Cryst.* **37**, 399–409. 10.1107/S0021889804005874 PMC2808709 20090869

[bb23] Shalom, G., Shaw, J. G. & Thomas, M. S. (2007). *Microbiology*, **153**, 2689–2699. 10.1099/mic.0.2007/006585-0 17660433

[bb24] Sheldrick, G. M. (2008). *Acta Cryst.* A**64**, 112–122. 10.1107/S0108767307043930 18156677

[bb25] Shneider, M. M., Buth, S. A., Ho, B. T., Basler, M., Mekalanos, J. J. & Leiman, P. G. (2013). *Nature (London)*, **500**, 350–353. 10.1038/nature12453 PMC3792578 23925114

[bb26] Silverman, J. M., Agnello, D. M., Zheng, H., Andrews, B. T., Li, M., Catalano, C. E., Gonen, T. & Mougous, J. D. (2013). *Mol. Cell*, **51**, 584–593. 10.1016/j.molcel.2013.07.025 PMC3844553 23954347

[bb27] Studier, F. W. & Moffatt, B. A. (1986). *J. Mol. Biol.* **189**, 113–130. 10.1016/0022-2836(86)90385-2 3537305

[bb28] Winn, M. D. *et al.* (2011). *Acta Cryst.* D**67**, 235–242.

[bb29] Winter, G. (2010). *J. Appl. Cryst.* **43**, 186–190.

[bb30] Winter, G. & McAuley, K. E. (2011). *Methods*, **55**, 81–93. 10.1016/j.ymeth.2011.06.010 21763424

[bb31] Yanisch-Perron, C., Vieira, J. & Messing, J. (1985). *Gene*, **33**, 103–119. 10.1016/0378-1119(85)90120-9 2985470

[bb32] Zhang, Z., Sauter, N. K., van den Bedem, H., Snell, G. & Deacon, A. M. (2006). *J. Appl. Cryst.* **39**, 112–119.

[bb33] Zheng, J. & Leung, K. Y. (2007). *Mol. Microbiol.* **66**, 1192–1206. 10.1111/j.1365-2958.2007.05993.x 17986187

[bb34] Zoued, A., Durand, E., Bebeacua, C., Brunet, Y. R., Douzi, B., Cambillau, C., Cascales, E. & Journet, L. (2013). *J. Biol. Chem.* **288**, 27031–27041. 10.1074/jbc.M113.499772 PMC3779704 23921384

[bb35] Zoued, A., Durand, E., Brunet, Y. R., Spinelli, S., Douzi, B., Guzzo, M., Flaugnatti, N., Legrand, P., Journet, L., Fronzes, R., Mignot, T., Cambillau, C. & Cascales, E. (2016). *Nature (London)*, **531**, 59–63. 10.1038/nature17182 26909579

[bb36] Zoued, A., Durand, E., Santin, Y. G., Journet, L., Roussel, A., Cambillau, C. & Cascales, E. (2017). *Bioessays*, **39**, 1600262. 10.1002/bies.201600262 28817192

